# Demystifying the Value of Minimal Clinically Important Difference in the Cardiothoracic Surgery Context

**DOI:** 10.3390/life13030716

**Published:** 2023-03-06

**Authors:** Dimitrios E. Magouliotis, Metaxia Bareka, Arian Arjomandi Rad, Grigorios Christodoulidis, Thanos Athanasiou

**Affiliations:** 1 Unit of Quality Improvement, Department of Cardiothoracic Surgery, University of Thessaly, Biopolis, 41110 Larissa, Greece; 2 Department of Anesthesiology, University of Thessaly, Biopolis, 41110 Larissa, Greece; 3 Department of Surgery and Cancer, Imperial College London, St Mary’s Hospital, London W2 1NY, UK; 4 Department of Surgery, University of Thessaly, Biopolis, 41110 Larissa, Greece

**Keywords:** cardiac surgery, thoracic surgery, minimal clinically important difference, MCID, quality of life, QoL, heart failure

## Abstract

The aim of this review is to describe the different statistical methods used in estimating the minimal clinically important difference (MCID) for the assessment of quality of life (QOL)-related and clinical improvement interventions, along with their implementation in cardiothoracic surgery. A thorough literature search was performed in three databases (PubMed/Medline, Scopus, Google Scholar) for relevant articles from 1980 to 2022. We included articles that implemented and assessed statistical methods used to estimate the concept of MCID in cardiothoracic surgery. MCID has been successfully implemented in several medical specialties. Anchor-based and distribution-based methods are the most common approaches when evaluating the MCID. Nonetheless, we found only five studies investigating the MCID in the context of cardiothoracic surgery. Four of them used anchor-based approaches, and one used both anchor-based and distribution-based methods. MCID values were very variable depending on the methods applied, as was the clinical context of the study. The variables of interest were certain QOL measuring questionnaires, used as anchors. Multiple anchors and methods were applied, leading to different estimations of MCID. Since cardiothoracic surgery is related to important perioperative morbidity, MCID might represent an important and efficient adjunct tool to interpret clinical outcomes. The need for MCID methodology implementation is even higher in patients with heart failure undergoing cardiac surgery. More studies are needed to validate different MCID methods in this context.

## 1. Introduction

One of the most important aspects of healthcare and perioperative management is the ability that physicians in general, and especially surgeons, have to understand, evaluate, and measure the effect of their interventions on patients by using a systematic approach. This necessity is higher when therapies intend to improve subjective outcomes, like the quality of life (QoL) metrics, thus making the complexity of evaluating the clinical value of therapeutic interventions even greater [1]. Nonetheless, the evaluation of variables that are highly subjective is always a challenging task given that an important difference may not represent a clinically significant change for either physicians or patients. Taking these facts into consideration, the minimal clinically important difference (MCID) term represents the concept of the smallest benefit as it is perceived by the individual patients [1] and represents a critical advance in measuring quality outcomes. MCID is a patient-centered concept and demonstrates not only the aspect of objective clinical improvement but also the importance that patients attribute to this change. The methodology associated with the MCID concept has been designed in a way that maximizes the provided information regarding the patient’s experience while preserving the clinical relevance to the patient-reported outcomes [1]. Given that there are certain challenges related to measuring significant differences in rare outcomes [2], several questionnaires [3,4,5] have been developed, necessitating the clinical interpretation of meaningful changes. Although these concepts are increasingly implemented in cardiothoracic surgery, the use of the MCID methods remains poor. The aim of the present study is to uncover the value of MCID implementation in cardiothoracic surgery and especially in high-risk patients such as those with reduced ejection fraction (EF). In the following paragraphs, we will provide a deep insight into all of the MCID concepts and techniques with real-life examples.

## 2. Materials and Methods

A thorough literature search was conducted through a systematic approach in three databases: (1) Pubmed (Medline), (2) Scopus (ELSEVIER), and (3) Google Scholar (last search date: 30 August 2022). Two independent reviewers (DEM, AAR) performed the literature search in these databases. The following terms were employed in all possible combinations: “minimal clinically important difference”, “mcid”, “cardiothoracic surgery”, “cardiac surgery”, “thoracic surgery”, “cardiothoracic surgery”, “vats”, “thoracotomy”, “coronary artery bypass grafting”, “cabg”, “valve surgery”, “valve replacement”, “heart failure”, “cardiac failure”, “hfref”, “hfpef”, “transcatheter aortic valve replacement”, “tavr”, and “quality of life”. Inclusion criteria were (1) original reports with ≥10 patients, (2) written in English, (3) published from 1980 to 2022, (4) conducted on human subjects, and (5) reporting outcomes of cardiothoracic surgery patients and patients with heart failure where MCID techniques were employed. Duplicate articles were excluded. The reviewers also hand-searched the reference lists of all included articles to identify additional potential studies. Two independent reviewers (DEM, AAR) extracted data from the studies that were included. Any discrepancies between the investigators about the inclusion or exclusion of studies were discussed with the senior author (TA) to incorporate only articles that best matched the criteria until a consensus was reached.

## 3. Results

### 3.1. The Rationale behind MCID Implementation

**Figure 1:**
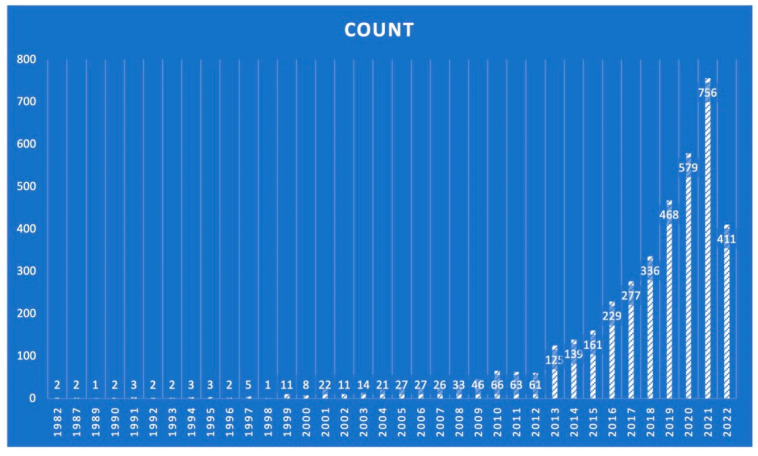
The total volume of articles on the topic of minimal clinically important difference (MCID) published per year according to PubMed/Medline.

**Figure 2:**
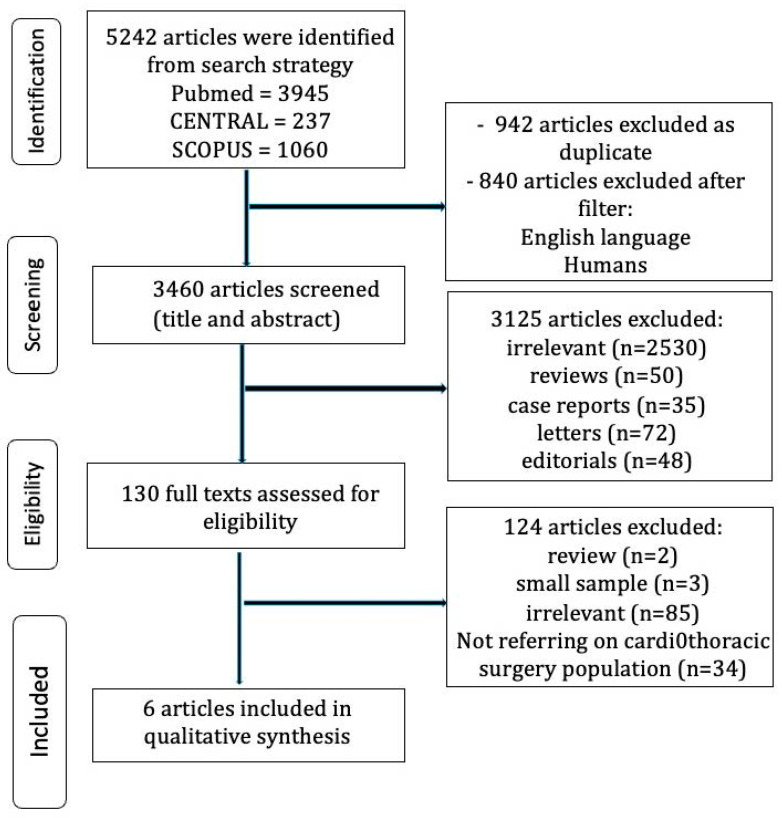
Trial flow presenting the literature search.

Recently, there has been wide implementation of MCID methodology in different medical specialties and fields, as is well presented in Figure 1 and Figure 2. The primary concept in favor of implementing the MCID methodology is that it enhances the clinical data interpretation, analysis, and assessment regarding the patient-reported changes following an intervention on the basis of whether the reported change is important and meaningful to individual patients. In fact, MCID methodology also facilitates all of the above functions in a feasible and appropriate manner. The direct consequence is that similar alterations on a numerical scale may not represent similar levels of clinical importance in different study samples. Furthermore, statistical importance is associated with the population size and its baseline attributes. Interestingly, in cases where the study sample is large, significant differences reported among groups might be marginal and not clinically relevant [6]. In this context, the MCID methodology has been developed as a response to these challenges.

### 3.2. Different Methodological Approaches for MCID

Cardiothoracic surgeons and cardiologists treat patients with several comorbidities in a variety of different settings. However, different settings create clinical questions and challenges of different natures that call for different approaches. In this context, the principles of MCID provide great value and therefore should be implemented. Below are the main methodological approaches to assess the minimum important difference (MID):

1. An important difference that represents a real alteration as perceived at the population or individual level.
2. A difference reflecting cost-effectiveness associated with healthcare institutions, perceived as systems.
3. A significant difference as perceived by patients in those cases where the interpretation of measures is not clear.
4. An important difference associated with a prognostic factor, to reach a reduction regarding an event of interest within a certain population.
5. A minimum of important differences that are perceived by individuals.

In addition, the above-described approaches affect the determination of the study sample size that should be enrolled to reliably evaluate the clinically important impact of medical or surgical intervention. Interestingly, the smaller the intervention effect is expected to be, the larger should be the required study population [7]. In the next paragraphs, we present the main methods to estimate MCID.

### 3.3. Distribution Methods Approach

The first method we describe herein is the distribution-based one. In fact, the distribution-based methodology is related except from the endpoints of interest also to the setting in which it is implemented. For example, these methods might differ corresponding to different interventions or study populations, where the variance is particularly homogenous [8]. These methods are mainly based on the distribution of the outcome scores, along with the heterogeneity reported among patients. Moreover, these estimates identify and assess the minimum rate of change that is necessary to demonstrate that the difference in an outcome is greater than what would be expected from plain chance [6]. Given that distribution-based estimates are not derived from individual patient assessments, preferably they should not be used to determine MCID. The concept behind their use is based on statistical reasoning since it can only identify a minimum detectable effect, that is, an effect that is unlikely to be attributable to random measurement error. The lack of an “anchor” linking these numerical scores to what is important for patients means that these methods fail to identify important and clinically meaningful outcomes for patients.

There are certain limitations related to the distribution methods. To begin with, this approach is primarily based on clear statistical reasoning. Consequently, it might identify a minimal detectable effect, not attributed to a random measurement error [9]. Consequently, the lack of anchoring relating the numeric estimates with an assessment of clinical significance limits the inherent potential of distribution-based methodology to identify clinically important outcomes for patients. In this context, “minimal detectable change” could replace the MCID term in cases where the difference is evaluated using distribution-based estimates [6]. Taking all of these into consideration, distribution-based methods are not recommended to be used as a first-line MCID measure.

### 3.4. Anchor-Based Approach

The anchor-based estimates represent the most frequently-used category of MCID methods. In fact, they allow for a comparison between the patient’s status indicated by an outcome estimate and an external criterion. Consequently, this is not a true external criterion but mainly represents the patient’s perception. This method then compares the changes between scores with an anchor question. For example, questions like “do you feel better after intervention?” are employed as a reference to determine if the patient’s clinical status has improved following treatment compared to baseline, based on the patient’s own experience. The clinical question used as an anchor must be friendly to answer, easily understandable, and relevant to the patients of interest. Commonly employed anchors might be scales assessing a presence of symptoms; a difference in health status; the severity of the disease; the treatment response; or the prognosis of future complications, morbidity, or mortality.

Responses referring to changes such as “somewhat/much better” represent high-value concepts given that they inform the researcher on the level of clinical improvement perceived by the patients. The most frequently used anchor is a measure associated with an established MID or implementing a patient’s subjective rating of change on a 5- or 7-Likert point scale [10]. Anchor-based methodology is characterized by the MCID approach by relating the perceived change with a numerical scale for a certain outcome. For instance, patients may be asked if they felt “pretty much the same,” “little”, or “quite better” following intervention. The next step is to “anchor” these responses (categorical variables) to the numerical measurement scale (continuous variable) used in the study. Furthermore, the implementation of MCID methodology for measuring functional status, as was described in the study by Hinman et al. [11], demonstrated that 75% of patients who reported a benefit (the anchor) also reported an enhancement similar or greater than the derived MCID. This evaluation of the level of change for the variable of interest using the MCID of the anchor is performed using linear or logistic regression statistical methods. For instance, the short-form-36 health survey (SF-36) questionnaire was validated in cardiothoracic surgical patients, using a logistic regression approach, to evaluate the independent risk factors for HRQOL deterioration at 6 months following surgery [12].

In those cases where the anchor represents a global level of change, this rating might be provided by the physician or the patient, but the existence of different perceptions of what demonstrates an important level of change might differ among them [13]. In addition, the anchor-based methodology has the important advantage of relating the demonstrated change to a certain score based on the patient’s experience. However, patients may attribute different values to a certain benefit (inter-patient variation), or the same patient may attribute a different value to the same benefit (intra-patient variation) at different time points, depending on the individual perceptions and circumstances, thus posing a certain bias and a level of heterogeneity [14]. Numerous decisions received in daily clinical practices regarding patient management are evaluated on the basis of the potential harm they might produce and are discussed in-depth during counseling.

One of the most critical aspects that should be taken into consideration when designing an anchor-based method remains the assessment of whether the demonstration of change is precise and easily understood. In fact, there have been identified four variations of the anchor-based methodology [15]: (1) the score change for each individual patient, (2) the score change among different patients, (3) the sensitivity/specificity evaluation methodology, and (4) the social comparison approach. In this context, the first methodology is based on patients’ ratings of their improvement [15]. The second approach employs sensitivity and specificity analyses. Sensitivity represents the proportion of patients reporting an absolute improvement with a score exceeding the threshold value. Specificity represents the number of patients reporting a deterioration, with a score lower than the threshold value or a truly negative outcome [16]. The third score implements the comparison between the patient response, as it is allocated in two distinct levels using a global scale. The fourth method represents the least popular one. In this context, the MID is derived by calculating the difference among patients by assessing their status as superior or inferior to other patients [15,17].

The main limitation of this approach is the anchor-related potential bias, given that it is based on a subjective assessment. For example, an anchor based on patients’ perception regarding their health status and the consequent post-intervention improvement might produce a recall bias [9]. On this basis, the validity of the anchor is crucial to demonstrate a reliable MCID. In addition, anchor-based approaches might be affected by the distribution and heterogeneity of scores within each category of the anchor. In fact, there are cases incorporating highly skewed data, thus affecting the MCID evaluation by outliers. Moreover, anchor-based estimates might be associated with an MCID derived from a unique subgroup of patients within a particular category of the anchor, thus leading to low-quality MCID calculations.

### 3.5. Consensus or Delphi Approach

Consensus or Delphi methods are based on the gathering of a panel of experts that provide independent opinions regarding the definition of a clinically relevant change. Panel members provide intellectual comments and suggestions in the provided opinions. The draft that is prepared with the sum of the opinions is revised again based on the members’ comments until a consensus is reached from the body of participating experts, and a numerical value is provided for the MCID. A recent article [11] provides an example of MCID technique implementation in pain evaluation using a scale that was developed based on a Delphi approach. Nonetheless, this approach is associated with a certain limitation. Given that the consensus methods are primarily based on experts’ opinions, rather than patients, to define the MCID, they might not represent a reliable method to evaluate what is a clinically important difference for a patient. Consequently, there is a certain bias that should be taken into consideration.

### 3.6. Potential Pitfalls When Implementing MCIDs

A relatively frequent phenomenon that has been highlighted by several researchers [11,18] is the smaller perceived change compared with the predefined MCID. In fact, this specific case is presented in circumstances where the patient sample is selected in order to achieve an augmented probability rate of identifying a benefit equal to the MCID, thus highlighting statistically significant changes even when the impact of an operation or clinical intervention is lower than the MCID [9]. In addition, another aspect regarding MCIDs that is important to comment on is the necessity to identify potential improvements derived from a surgical operation, endoscopic or clinical intervention associated with morbidity/mortality, and financial charges. Consequently, it remains always a high priority to have a wide (360°) view of all aspects of care, from first diagnosis, counseling, and admission to discharge, regardless of whether there have been favorable or unfavorable events.

Based on the previous assumptions, it is clear that there is an armamentarium of alternative approaches to deriving an MCID. Nonetheless, it remains important for the physician/surgeon/scientist conducting or reading an MCID report to know the exact way it was calculated and assessed. As we have already highlighted, not every MCID method corresponds to a particular context. Moreover, it is often challenging to distinguish between the terms MID and MCID. To face this challenge, an article [19] has been proposed that MIDs should be divided into three distinct subcategories, using a suffix following the term “MID”: MID-S (MID–statistical), MID-C (MID–clinical outcome), and MID-P (MID–patient determined). Nonetheless, special caution is necessary when merging different MCID methods. Finally, whichever MCID approach is chosen and used, it represents an aiding tool for the interpretation of patient outcomes derived from clinical interventions and as such should be treated. The clinical context, the physician’s experience, and institutional arrangements should always be taken into consideration.

## 4. Discussion

### 4.1. MCID Implementation in Cardiothoracic Surgery

To assess the level of implementation of MCID methodology in cardiothoracic (CT) surgery, six studies were identified through the thorough literature search and were included that employed the MCID methodology to assess QoL in those patients (Table 1) [12,20,21,22,23,24]. The first study employed anchor-based methods to calculate MCID metrics [12]. According to that study, a statistically important difference was demonstrated in the perioperative QoL scores [12]. However, the level of change was not statistically significant according to the assessment with MCID techniques [12]. This case represents an important and useful example of how a seemingly statistically important change might not be clinically important when assessed through an MCID approach, and this is the real value of the MCID methodology.

Another study [20] evaluated the association of surgical aortic valve replacement (SAVR) and QoL, along with the variance with age, especially for high-risk patients. This was an observational, multicenter cohort study that was conducted in accordance with the RECORD guidelines (Reporting of studies Conducted using Observational Routinely collected Data) [25]. In this study, the authors used the SF-36 questionnaire in an anchor-based approach context to evaluate the MCID regarding the post-aortic valve replacement QoL. Based on an MCID of five points, the authors estimated an increase (>5), decrease (≤5), or no difference regarding QoL for each individual patient [20]. Sensitivity analysis were also performed using an MCID that incorporated four points [20].

Furthermore, in another study [21] researchers evaluated QoL in patients with severe aortic stenosis undergoing surgery or conservatively treated, using the SF-36 and EQ-5D questionnaires in order to assess QoL in an anchor-based context (Table 1). This study showed a significant improvement in QoL in patients with severe aortic stenosis undergoing SAVR [21]. Furthermore, in their study, Rinaldo et al. [22] provide another useful example of successful MCID implementation. To evaluate the functional capacity in post-acute cardiac patients, they used the patient global rating of change in an anchor-based approach. The rationale behind their choice was based on its suitability for assessing the perception of change from the individual patient’s perspective [26]. In fact, the authors adopted the anchor-based method and the patient global impression of change (PGIC) as an anchor. The format for the assessment included the patients’ interview prior to discharge, where they described the perceived change of health status and physical performance using a seven-point Likert scale [27]. They found that an MCID >1 point of change, thus providing a reference value that could serve as an explicit goal for rehabilitation interventions and the monitoring of functional status.

**Figure 3:**
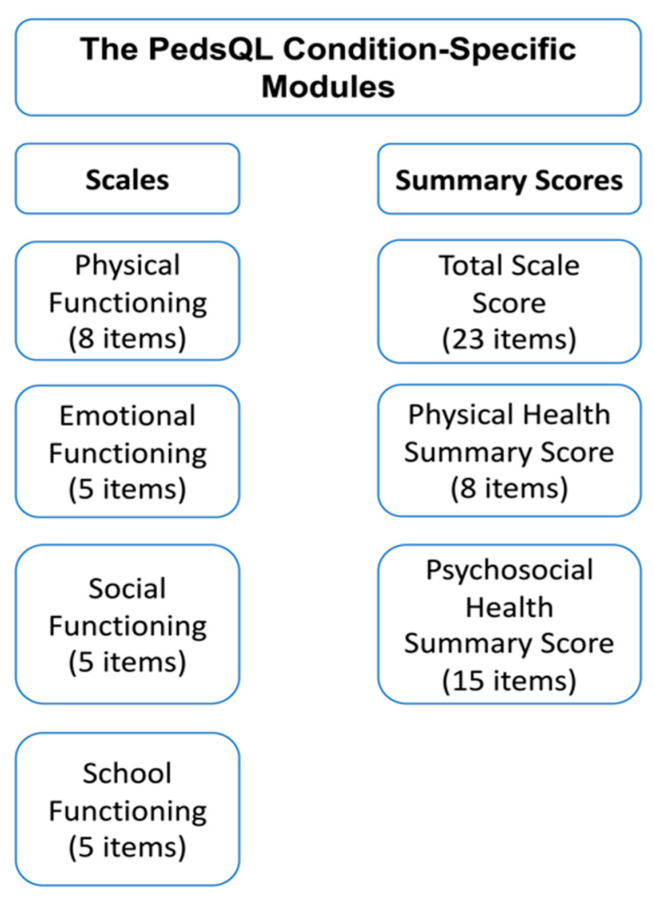
The structure of PedsQL as an example of an anchor in the minimal clinically important difference (MCID) context.

In addition, in their study, Meenaghan et al. [23] described their experience of implementing the MCID approach to measure health-related QoL for pediatric cardiac patients after extracorporeal life support. This is an interesting study, given that it implements an MCID approach in a very distinctive population: children. In fact, in this study, the authors used the pediatric quality of life inventory (PedsQL) questionnaire using an anchor-based methodology [27] to assess the health-related QoL. The structure of PedsQL is presented in Figure 3 as an example of an anchor. A 5-point Likert scale was used and filled across by both the child and parent and then was analyzed by physicians. According to their findings, the extracorporeal life support group demonstrated a lower health-related quality of life score than the control group.

Finally, Dahlberg et al. [24] investigated the implementation of an MCID approach in patients undergoing therapeutic thoracentesis. They used the visual analogue scale (VAS) to measure changes in discomfort and dyspnea related to pleural interventions. In fact, discomfort and dyspnea represent common morbidities related to and following pleural interventions. Consequently, it is important to assess QoL from an MCID point of view. To evaluate the MCID, they employed an anchor-based method, using a Likert scale [24]. In fact, patients were asked to identify their level of perceived chest discomfort on VAS before, during, and after the procedure. Five minutes later, patients were asked to indicate their overall level of chest discomfort on VAS, followed by a seven-point Likert scale, with the following options: (a) large/moderate improvement, (b) small but worthwhile improvement, (c) slight but not worthwhile improvement, (d) no change, (e) slight but not significant increase in discomfort, (f) small but significant increase in chest discomfort, and (g) large/moderate increase in discomfort. This study demonstrated that the MCID concept can be well-implemented in the management of chest discomfort as an important patient-centered clinical tool during pleural procedures.

### 4.2. MCID Implementation in the Context of Heart Failure and Transcatheter Aortic Valve Replacement (TAVR)

The MCID concept is suitable, especially for high-risk, multimorbid patients with a significantly depleted quality of life. One such group includes those patients suffering from heart failure. In fact, there are certain studies that have implemented MCID methods in order to better assess outcomes in patients with heart failure. The value of MCID approach is highly relevant in such patients, given that QoL is really important in that group of patients and measuring it is really important [28,29]. In the next paragraphs, we will present certain articles as examples of MCID method implementation in patients with heart failure.

To begin with, in a recent research letter Jain et al. [29] aimed to define an MCID in a 6-min walk test for patients with heart failure and mitral valve disease. In this context, they used the Kansas City Cardiomyopathy Questionnaire-Overall Summary score (KCCQ-OS) along with the 6-min walk distance (6MWD) to assess health and functional status. In fact, 6MWD was used as an anchor to KCCQ-OS, and that is another example of an anchor-based approach to MCID. According to their findings, they suggested that approximately a 25-m improvement in 6MWD may represent a clinically meaningful change from the perspective of the individual patient. However, there was no significant change in 6MWD among patients who experienced small but clinically meaningful changes in KCCQ, thus suggesting that patients may experience a modest improvement in self-perceived health status without an objective improvement in functional status.

KCCQ was also used in another study that assessed the MCID in the health status of patients with heart failure with preserved ejection fraction (HFpEF) versus heart failure with reduced ejection fraction (HFrEF) [30]. In this study, a patient global impression of severity (PGIS) questionnaire was administered to patients and was used as an anchor to KCCQ. The authors demonstrated that a change in KCCQ-TSS of >9 points in HFrEF and >7 points in HFpEF patients represents the MCID for improvement [30]. Finally, another study used an anchor-based approached to assess the MCID for 6MWD in patients with HF and iron deficiency [31]. In this study, the patient global assessment (PGA), a health-related QoL tool based on a Likert scale, was administered to patients and was used as a clinical anchor [31]. The authors demonstrated that the MCID for improvement in exercise capacity on the 6MWT was 14–15 m in patients with HFrEF and iron deficiency.

All of these articles represent real-life examples of the important value that MCID provides for the deeper interpretation and evaluation of study data, especially in high-risk patients such as those with HF. Perhaps further studies are necessary to implement these methods in patients with HF undergoing cardiothoracic surgery.

The TAVR procedures represent another field of potential interest for MCID method implementation. In fact, there has been a strong focus and investigation on the impact of TAVR on QoL of patients [32]. The studies that assessed the effect of TAVR on QoL mainly used patient-reported outcome measures (PROMS) [33]. In this context, the implementation of an MCID approach is crucial to define the least clinically relevant change. Given that there is an important debate comparing SAVR versus TAVR procedures underway, the potential role of MCID is important. In fact, the value of these approaches is even higher in multimorbid or high-risk patients, such as those patients with HF undergoing TAVR for aortic regurgitation following left ventricular assist device implantation [34]. Nonetheless, through our literature search we could not identify any articles using MCID methods for patients undergoing TAVR.

## 5. Conclusions

In the current review, we tried to investigate, describe, and summarize the fundamental principles and methodology associated with MCID. In this context, the MCID approach employs the smallest difference in score in any outcome of interest that patients can perceive as beneficial or the opposite. Consequently, MCID helps to take clinical decisions by highlighting the superiority of patient perceptions while also being used as a crucial tool for the evaluation of the sample size of each study. Nonetheless, MCID represents a highly heterogeneous concept and methodology, given the several alternative methods employed for its calculation. These different methods can potentially generate differential estimates regarding a health situation or disease limited in creating universally comparable or useful values of health benefit/harm perceptions. Given that cardiothoracic surgery is mainly associated with high-risk, multi-morbid patients and significant perioperative morbidity, it represents a surgical field where MCID might be a valuable tool to interpret clinical outcomes. Nonetheless, validating different MCID approaches in the cardiothoracic surgery context is necessary. Finally, there are certain limitations in the present study, associated with the small number of included studies and the lack of any randomized controlled trials.

## Figures and Tables

**Table 1:** Studies implementing minimal clinically important difference (MCID) methodology in cardiothoracic surgical interventions.

Study ID	Country of Origin	Study Population	MCID Methodology	Variables	Outcomes
Grand 2018 [12]	France	326	Anchor-based	Short-form survey SF-36 questionnaire	Post-cardiac surgery quality of life (QoL) improvement
Blokzijl 2021 [20]	Multinational	899	Anchor-based	SF-36 questionnaire	Post-AVR (aortic valve replacement) QoL improvement
Auensen 2018 [21]	Norway	442	Distribution- and anchor-based	EQ-5D and SF-36 questionnaires	Post-AVR QoL enhancement
Rinaldo 2022 [22]	Italy	392	Anchor-based	SPPB test; patient global impression of change was used as an anchor	Post-cardiac surgery rehabilitation QoL improvement
Meenaghan 2021 [23]	Ireland	41	Anchor-based	PedsQL questionnaire	QoL improvement in pediatric cardiac patients after extracorporeal life support
Dahlberg 2020 [24]	USA	262	Anchor-based	VAS	Chest discomfort in patients undergoing therapeutic thoracentesis

Abbreviations: QoL: quality of life; SF-36: short form-36 health survey; PedsQL: pediatric quality of life inventory; SPPB: short physical performance battery; and VAS: visual analogue scale.

## Data Availability

All of the associated data are available by the authors upon request.
